# Effect of peritoneal dialysis in end-stage renal disease on apixaban pharmacokinetics

**DOI:** 10.1093/ndt/gfad087

**Published:** 2023-05-11

**Authors:** Laure Peyro-Saint-Paul, Clémence Bechade, Alexandre Cesbron, Danièle Debruyne, Marie Brionne, Sylvie Brucato, Mélanie Hanoy, Audrey Dumont, Anais R Briant, Jean-Jacques Parienti, Thierry Lobbedez, Maxence Ficheux

**Affiliations:** CHU de Caen Normandie, Department of Clinical Research and Biostatistics, Caen, France; Department of Nephrology, Normandie University, UNICAEN, CHU de Caen Normandie, Caen, France; ANTICIPE, U1086 INSERM-UCN, Centre François Baclesse, Caen, France; CHU de Caen Normandie, Pharmacologie, Caen, France; CHU de Caen Normandie, Pharmacologie, Caen, France; CHU de Caen Normandie, Hématologie Biologique, Caen, France; CHU de Caen Normandie, Centre de Recherche Clinique, Caen, France; CHU Rouen, Nephrology, Dialysis and Kidney Transplantation, Rouen, France; Univ Rouen Normandie, INSERM EnVI U1096, “Endothelium, Valvulopathy and Heart Failure”; CHU Rouen, CIC-CRB 1404, Department of Pharmacology, Rouen, France; Department of Clinical Research and Biostatistics, CHU de Caen Normandie and Caen Normandy University, Caen, France; Department of Clinical Research and Biostatistics, CHU de Caen Normandie and Caen Normandy University, Caen, France; INSERM U1311 DYNAMICURE, Caen Normandy University, Caen, France; Department of Nephrology, Normandie University, UNICAEN, CHU de Caen Normandie, Caen, France; ANTICIPE, U1086 INSERM-UCN, Centre François Baclesse, Caen, France; CHU de Caen Normandie, Néphrologie, Caen, France

Limited pharmacokinetics data are guiding the use of apixaban in end-stage renal disease (ESRD) patients on hemodialysis but none on peritoneal dialysis. Apixaban could be an alternative to harmful warfarin anticoagulation [1, 2]. Before conducting studies to explore the efficacy and safety of apixaban in this patient population, a pharmacokinetic study was required which is essential to determine the appropriate dosage [3]. The objective of the ApiDP (‘APIxaban et Dialyse Péritonéale’) study was then to assess the effect of peritoneal dialysis in ESRD patients on the pharmacokinetic parameters of apixaban. ApiDP was a prospective, controlled pharmacokinetics trial (NCT04006093) which included participants in two French University hospitals. Each ESRD patient on peritoneal dialysis was matched to a volunteer with normal renal function based on age, weight and sex to act as control. A single oral 5 mg dose of apixaban was administered and adequate blood and urine ± dialysate samples were collected for determination of pharmacokinetic parameters during 72 h. ESRD patients were undergoing continuous ambulatory peritoneal dialysis at enrollment and the peritoneal dialysis scheme was standardized at the time of apixaban administration: patients received four exchanges per day with dialysate volumes of 2 L per exchange: Dianeal^®^ (6 h), Nutrineal^®^ (6 h), Physioneal 40: 1.36%^®^ (4 h) and Extraneal^®^(8 h) solutions. The pharmacokinetic parameters of apixaban were derived from plasma concentration–time curve using a 1-compartment open model. The apparent first-order rate constant (ke) was determined by least squares regression analysis of the terminal phase of the plasma concentration–time curves. The resulting parameters were calculated as follows: apparent half-life (T_1/2_) from the relation T_1/2_ = Ln2/ke; apparent volume of distribution (V/F) from D/C_0_ where D is the administered dose and C_0_ the plasma concentration extrapolated at time 0; AUC_0__-inf_, the area under the concentration–time curve extended to infinity from AUC_0__-inf_ = AUC_0-24 h_ + (C_24_h/ke), AUC_0-24 h_ being calculated using the linear trapezoidal rule over the interval of 0 to 24 h; apparent total plasma clearance, ratio of total plasma clearance on bioavailability (Cl/F) from the equation Cl/F = D/AUC_0__-inf_. The maximum plasma concentration (C_max_) and the time required to reach C_max_ (T_max_) were observed from the concentration–time profile for each participant. The peritoneal dialysis extraction ratio (PDER) (fraction of apixaban in the dialysis solution) was estimated for the first 24 h period using the following equation: PDER(%) = (Dd/D) × 100, where Dd is the total amount of drug recovered in the peritoneal dialysis fluid at the end of the 24 h period following apixaban administration. The total amount of apixaban recovered in urine (Du) and the renal extraction ratio (RER) were so determined for the first 24 h period. Renal and peritoneal clearances (Cl_r_ and Cl_p_, respectively) were calculated during the 24 h period that followed apixaban administration using the following relations: Cl_r_ = Q_24h_ urine/AUC_0-24 h_ and Cl_p_ = Q_24h_ dialysate/AUC_0-24 h_.

**Figure 1:**
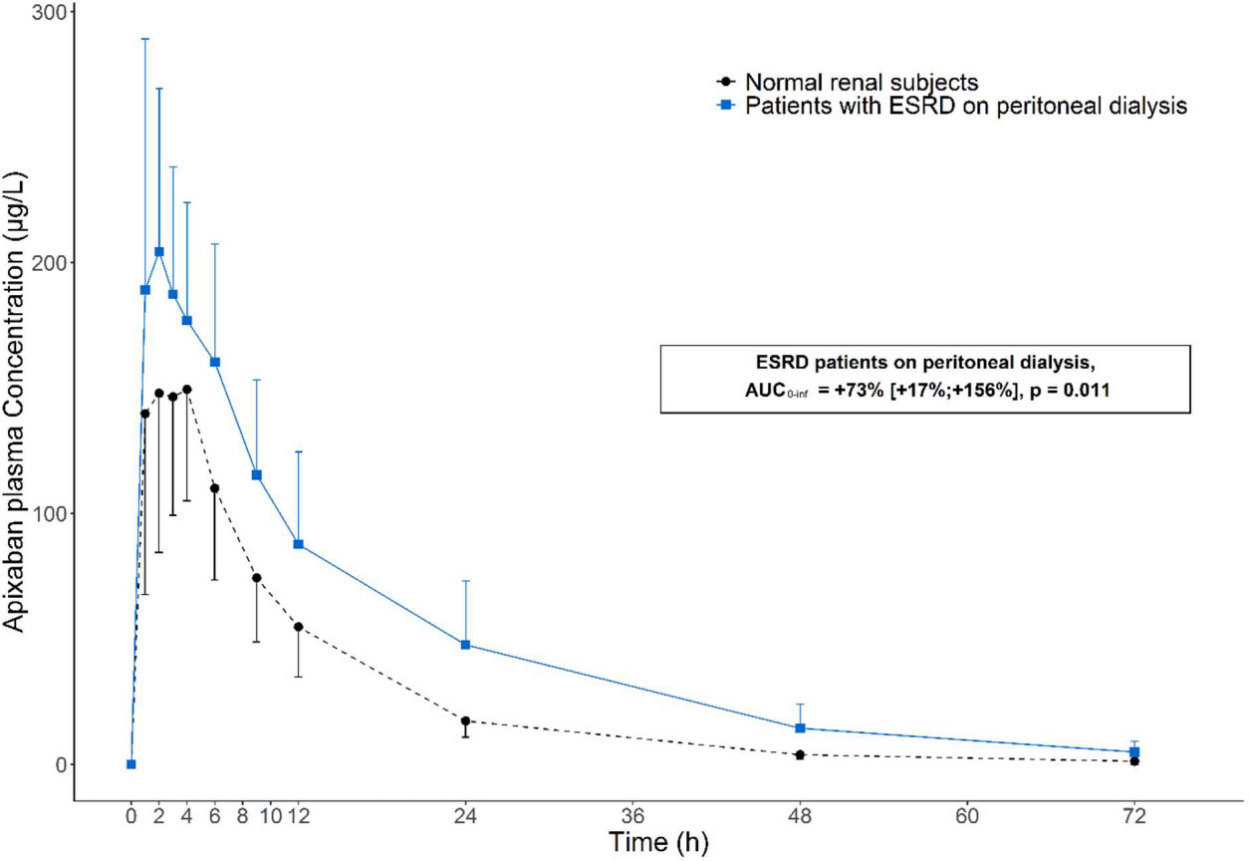
Linear mean (±SD) plasma concentration–time profiles of apixaban by renal function group following a single 5-mg apixaban dose.

Twenty-four subjects were included: 12 subjects with ESRD on peritoneal dialysis and 12 matched control volunteers. The mean [± standard deviation (SD)] age was 63 (±9) years old. Sixteen subjects were men, eight were women. Their mean body weight was 74 (±13) kg. Among peritoneal dialysis patients, all had a mean urine output of 1180 (±480) mL/24 h and a mean renal clearance of endogenous creatinine and urea (UV/P) of 2.27 (±1.31) mL/min. Accumulated time since peritoneal dialysis initiation at the time of study enrollment was 22 (±16) months. Volunteers had a mean estimated glomerular filtration rate (Chronic Kidney Disease Epidemiology Collaboration) of 88 (±10) mL/min. Figure 1 illustrates the kinetics of apixaban plasma concentrations in 12 patients and 12 healthy volunteers administered with a single oral dose of apixaban (5 mg). Concentrations peaked and then declined in a mono-exponential manner until 48 h, as demonstrated by the correlation coefficient of the linear regression data, Ln C = f(t), which obtained 0.983 ± 0.022 (mean ± SD) and 0.987 ± 0.008 in ESRD patients and controls, respectively. Table 1 describes pharmacokinetic parameters. In patients, the geometric mean C_max_, T_max_ and V/F values did not significantly differ from those of healthy group. However, compared with controls, apixaban AUC_0__-inf_ and T_1/2_ from ESRD patients on peritoneal dialysis were significantly higher: +73% (17–156) (*P* = .011) and +40% (24–58) (*P* < .001), respectively. Apparent Cl/F varied in line and was 40% lower (*P* = .016). Renal and peritoneal clearances, 1.0 ± 0.1 mL/min and 0.2 ± 0.1 mL/min, respectively, were negligible compared with the 12.4 ± 0.7 mL/min renal clearance determined in volunteers. In this first pharmacokinetics study of apixaban in patients with ESRD on peritoneal dialysis, we demonstrated differences in pharmacokinetic parameters in comparison with a normal renal function population. The AUC_0__-inf_ and T_1/2_ were significantly higher in subjects with ESRD undergoing peritoneal dialysis compared with healthy subjects. Clearance was reduced due to limited renal elimination and the passage in the dialysis peritoneal solution was very low. The robustness of the results of our study was further demonstrated by the consistency of the pharmacokinetic parameters in the group of healthy volunteers in comparison with other reports in healthy subjects [4]. In comparison with the data from the four pharmacokinetics studies on hemodialysis, apixaban is demonstrated to be less dialyzable by the peritoneal rather than the hemodialysis technique [5–8]. One might argue that the greater residual kidney function of peritoneal dialysis patients as well as the continuous purification provided by peritoneal dialysis may favor the renal elimination of apixaban; however, this is not the case. Our study supports the cautious reduction of apixaban dose from 5 to 2.5 mg twice daily for ESRD patients on peritoneal dialysis, subject to further pharmacokinetics/pharmacodynamics studies and clinical trials.

**Table 1:** Pharmacokinetic parameters in patients with ESRD on peritoneal dialysis compared with normal renal subjects.

	Patients with ESRD on peritoneal dialysis (*n* = 12)	NR subjects (*n* = 12)	Ratio (ESRD/NR) (95% CI)	*P*-value^a^
T_1/2_ (h), geometric mean ± SE	11.4 ± 0.7	8.1 ± 0.2	1.40 (1.24–1.58)	**<.001**
ke (h^–^^1^), geometric mean ± SE	0.061 ± 0.004	0.085 ± 0.002	0.71 (0.63–0.80)	**<.001**
V/F (L), geometric mean ± SE	24.1 ± 2.5	28.8 ± 2.6	0.83 (0.63–1.11)	.19
T_max_ observed (h), geometric mean ± SE	1.6 ± 0.2	2.0 ± 0.3	0.77 (0.46–1.31)	.31
C_max_ observed (µg/L), geometric mean ± SE	214.6 ± 23.9	171.8 ± 16.0	1.25 (0.90–1.73)	.17
AUC_0-24_ (h × µg/L), geometric mean ± SE	2391.0 ± 269.6	1607.6 ± 137.4	1.49 (1.06–2.09)	**.027**
AUC_0-inf_ (h × µg/L), geometric mean ± SE	3114.7 ± 404.3	1802.7 ± 159.1	1.73 (1.17–2.56)	**.011**
Cl/F (mL/min), geometric mean ± SE	28.3 ± 3.3	46.7 ± 3.3	0.60 (0.41–0.89)	**.016**
% administered dose in 24 h urine, geometric mean ± SE	2.80 ± 0.45	23.90 ± 1.27	0.12 (0.08–0.17)	**<.001**
% administered dose in 24 h dialysate, geometric mean ± SE	0.67 ± 0.16	NA	NA	NA

a *P*-value obtained using paired Student's *t*-test with lognormal distribution.

Bold p-value denotes statistical significance.

NR, normal renal; SE, standard error; NA, not analysable; T_1/2_, half-life; ke, elimination constant; V/F, apparent volume of distribution; T_max_, observed time to peak; C_max_, maximum observed plasma concentration; AUC_0-24_, area under the plasma concentration–time curve from time 0 to time 24 h; AUC_0-inf_, area under the plasma concentration–time curve from time 0 extrapolated to infinite time; Cl/F, apparent total plasma clearance.
